# Dicentrine Purified from the Leaves of *Ocotea puberula* Controls the Intracellular Spread of *L. (L.) amazonensis* and *L. (V.) braziliensis* Amastigotes and Has Therapeutic Activity as a Topical Treatment in Experimental Cutaneous Leishmaniasis

**DOI:** 10.3390/microorganisms13020309

**Published:** 2025-01-30

**Authors:** Jéssica Adriana Jesus, Gabriela Venicia Araujo Flores, Dalete Christine da Silva Souza, Daniela Costa Tristão, Dolores Remedios Serrano, Aikaterina Lalatsa, Márcia Dalastra Laurenti, João Henrique Ghilardi Lago, Humberto Gomes Ferraz, Rosana Pereira da Silva, Luiz Felipe Domingues Passero

**Affiliations:** 1 Institute of Biosciences, São Paulo State University (UNESP), Praça Infante Dom Henrique, s/n, São Vicente 11330-900, SP, Brazil; jessica.dolly@hotmail.com (J.A.J.); gabyaraflo@gmail.com (G.V.A.F.); 2 Institute for Advanced Studies of Ocean, São Paulo State University (UNESP), Rua João Francisco Bensdorp, 1178, São Vicente 11350-011, SP, Brazil; 3 Laboratory of Pathology of Infectious Diseases (LIM50), Department of Pathology, Medical School, São Paulo University, São Paulo 01246-903, SP, Brazil; mdlauren@usp.br; 4 Center for Natural and Human Science (CCNH), Federal University of ABC, Santo André, São Paulo 09210-580, SP, Brazil; christinesilvax@gmail.com (D.C.d.S.S.); danielatristao@gmail.com (D.C.T.); joaohglago@gmail.com (J.H.G.L.); 5 Department of Pharmaceutics and Food Science, Faculty of Pharmacy, Universidad Complutense of Madrid, Plaza Ramon y Cajal s/n, 28040 Madrid, Spain; drserran@ucm.es; 6 CRUK Formulation Unit, School of Pharmacy and Biomedical Sciences, University of Strathclyde, John Arbuthnot Building, Robertson Wing, 161 Cathedral St, Glasgow G4 0RE, UK; aikaterini.lalatsa@strath.ac.uk; 7 Pharmaceutical Development and Innovation Laboratory (Deinfar), Department of Pharmacy, School of Pharmaceutical Sciences, University of Sao Paulo, Professor Lineu Prestes Avenue, Sao Paulo 05508-580, SP, Brazil; sferraz@usp.br (H.G.F.); rosanapesi@alumni.usp.br (R.P.d.S.)

**Keywords:** *Ocotea puberula*, *Leishmania (Leishmania) amazonensis*, *Leishmania (Viannia) braziliensis*, treatment

## Abstract

Leishmaniasis, a complex disease caused by protozoal parasites of the genus *Leishmania*, presents various clinical forms, particularly a cutaneous clinical form. Treatment is typically performed with pentavalent antimonial and amphotericin B, both of which have severe side effects that hinder patient compliance. This emphasizes the need for the development of new, effective, and safe treatments. In this study, the leishmanicidal activity of the methanolic extract, an alkaloid-enriched fraction and dicentrine, the main alkaloid of the leaves of *Ocotea puberula* (Lauraceae), a native Brazilian plant traditionally used by the indigenous population to treat skin affections, was investigated *in vitro*. Additionally, an *in vivo* study evaluated the efficacy of a topical cream containing 0.5% dicentrine. The *in vitro* studies demonstrated high activity and selectivity of methanolic extract, alkaloid-enriched fraction, and dicentrine against the promastigote and amastigote forms of *Leishmania (Leishmnia) amazonensis* and *Leishmania (Viannia) braziliensis*. The leishmanicidal effect of dicentrine was related to the modulation of macrophage microbicidal activity. A cream containing 0.5% dicentrine showed high stability and, in permeation studies, dicentrine was retained in a skin-mimicking artificial membrane. This cream effectively inhibited the progression of the skin lesion in BALB/c mice infected with *L. (L.) amazonensis*, together with a reduced parasite number. Thus, dicentrine offers a promising alternative to the treatment of skin leishmaniasis.

## 1. Introduction

Leishmaniasis is considered one of the most important neglected tropical diseases (NTD) in the world and is caused by at least 20 parasitic protozoal species, most of them prevalent in Latin America [1]. Two main clinical forms can be recognized in humans, such as cutaneous and visceral leishmaniasis. In Latin America, cutaneous leishmaniasis (CL) can be further classified as localized cutaneous leishmaniasis (LCL), mucocutaneous leishmaniasis (ML), and diffuse anergic cutaneous leishmaniasis (ADCL) [2]. In Brazil, two main parasitic species have significant epidemiological importance, such as *Leishmania (Leishmania) amazonensis*, which is the etiologic agent of CL and ADCL, and *Leishmania (Viannia) braziliensis* that causes CL and ML [3,4]. Globally, it is estimated that more than 1 billion people live in endemic regions of leishmaniasis and are at risk of infection. Furthermore, around 1 million new cases of CL occur annually [5].

Although the clinical forms of leishmaniasis differ from each other, WHO treatment is based on pentavalent antimonials and amphotericin B [6]. Pentavalent antimonial was originally developed as an emetic agent but has been used in therapy as a first-line drug since 1940 [7]. Pentavalent antimonials are administered intravenously or intramuscularly and are associated with local and systemic side effects, including nausea, weakness, cardiotoxicity, hepatotoxicity, and pancreatitis [8], in part due to the low selectivity for humans. In some geographical locations, such as Bihar (India), the emergence of parasite resistance to antimonials is demonstrated by the fact that around 60% of patients were refractory to antimonial treatment [9,10]. Amphotericin B is the main second-line treatment for leishmaniasis, especially in cases associated with unmanageable symptoms in the patient or antimonial failure [11]. Amphotericin B has selectivity to ergosterol (non-mammalian cells) but also binds cholesterol present in the cell membrane of mammalian cells, causing severe side effects such as fever, chills, arthralgia, nausea, vomiting, headache, and nephrotoxicity [12]. In contrast, liposomal amphotericin B, such as Ambisome^®^, causes low toxic events in patients while maintaining efficacy compared to amphotericin B [13]. Additionally, safer formulations of amphotericin B (liposomal forms) are refractive in terms of cost in low-income countries [14]. Other drugs, such as miltefosine, pentamidine, and paromomycin, can be used as second-line treatments for leishmaniasis. However, significant concerns arise due to their high costs and variable efficacy, which can differ based on geographical location and specific infecting species [15,16].

None of these drugs were specifically developed for leishmaniasis, and therefore lack selectivity, which correlates with severe side effects in patients [17]. Therefore, it is urgent to develop new and safe alternative drugs for the treatment of patients with leishmaniasis. Medicinal plants represent valuable resources to find highly selective bioactive compounds for leishmaniasis [18,19]. One of these plants is *Ocotea puberula* (Rich.) Nees (Lauraceae) that has a wide distribution in Brazil [20], and according to phytochemical studies, this plant accumulates different metabolites, such as the alkaloids dicentrine [21], ocoteine [22], dehydroocoteine, and didehydroocoteine [23], which account for the pharmacological activity of this plant species, such as antinociceptive, anti-inflammatory, antimycobacterial, and antiparasitic effects [24,25,26,27,28].

Furthermore, *O. puberula* has been traditionally used by indigenous populations in southern Brazil for the treatment of skin disorders [29], indicating its potential application in leishmaniasis. While several studies have demonstrated the anti-tumor properties of *O. puberula* and the dicentrine alkaloid [30], as well as their effectiveness against *Trypanosoma cruzi* [31], only one study has revealed that dicentrine exhibits anti-*Leishmania (Leishmania) infantum* activity [32]. However, to our knowledge, no analysis has been performed on the efficacy of *O. puberula* and dicentrine against *Leishmania* species responsible for cutaneous leishmaniasis. Furthermore, *in vivo* studies revealed that the components of *O. puberula*, such as bark mucilage and pure dicentrine, are not toxic to experimental animals [33,34,35]. These studies suggested that the components of *O. puberula*, such as dicentrine, can be considered interesting alternatives to develop new drugs for NTDs, such as leishmaniasis.

The present work shows that an extract and an alkaloid-enriched fraction purified from the Brazilian plant *Ocotea puberula* were active on promastigote and amastigote forms of *L. (L.) amazonensis* and *L. (V.) braziliensis* with higher selectivity compared to miltefosine. Furthermore, the major alkaloid, dicentrine, present in the alkaloid fraction was shown to present immunomodulatory activity in macrophages that was correlated with the leishmanicidal effect *in vitro* and *in vivo*. Thus, dicentrine may be a viable alternative for the treatment of leishmaniasis.

## 2. Materials and Methods

### 2.1. General

The NMR spectra were recorded on a Varian INOVA 500 (Varian, Palo Alto, CA, USA) spectrometer, using CDCl3 as the solvent and TMS as the internal standard. The HR-ESIMS spectra data were obtained on a Bruker Daltonics (Billerica, MA, USA) q-TOF Maxis 3G spectrometer operating on electrospray ionization in positive mode. Silica gel 60 (230–400 mesh) and Sephadex LH-20 (Sigma-Aldrich, St. Louis, MO, USA) were used for column chromatography, while silica gel F254 (Merck, Rahway, NJ, USA) was used for thin-layer analytical chromatography.

### 2.2. Plant Material

The *O. puberula* plant was collected in São Paulo, SP, Brazil, during June, 2020. Plant identification was performed by M.Sc. Guilherme M. Antar; a voucher specimen was compared with that previously deposited in the Herbarium of the Institute of Biosciences, University of São Paulo, SP, Brazil (Antar 1031) [27].

### 2.3. Extraction and Isolation Procedures

The leaves of *O. puberula* were dried at 40 °C for 72 h. The plant material (300 g) was defatted with hexane and extracted using methanol until exhaustion (8 × 1 L). The crude methanolic extract produced was subjected to evaporation under reduced pressure, yielding 28 g of methanolic extract. The dried material was resuspended in dichloromethane and extracted with 1% HCl (pH2). The aqueous phase was collected and treated with ammonium hydroxide until pH 10 and extracted with dichloromethane (3 × 300 mL). The organic phase was dried over sodium sulfate, and the solvent was evaporated under reduced pressure to afford the alkaloid-enriched fraction that yielded 1.8 g, constituted by dicentrine, dicentrine-β-N-oxide, dehydrodicentrine, predicentrine, N-methyllaurotetanine, and cassythicine as previously demonstrated [27]. This fraction was subjected to silica gel column chromatography using mixtures of the solvents EtOAc (ethyl acetate):MeOH (methanol) (1:9, 8:2, 6:4, 1:1, 4:6, 2:8, and 1:9) to obtain dicentrine (purity > 99%) as an amorphous powder, yielding 0.56 mg [27]. Other compounds were not purified due to low yield (0.001–0.004%).

### 2.4. Animals and Bone Marrow-Derived Macrophages

This study was carried out according to the recommendations of the Guide for the Care and Use of Laboratory Animals of the Brazilian National Council for Animal Experimentation. The protocol was approved by the Ethics Committee on Animal Experiments of the Institutional Animal Care and Use Committee of the Faculty of Medicine of São Paulo University (CEUA1648/2022). Eight-week-old male BALB/c mice were obtained from the Animal Facility Center of the Medical School, São Paulo University. Before the experiments, the animals were anesthetized with ketamine (100 mg/kg) and xylazine (10 mg/kg), followed by a lethal dose of sodium thiopental (150 mg/kg).

The bone marrows were obtained from the femurs and tibias of 8-week-old BALB/c mice using Hanks balanced salt solution (HBSS). After three wash steps at 800× *g*, for 7 min at 4 °C, the red blood cells were lysed using lysing buffer (0.17 M NH4Cl, pH 7.4) for 7 min at 4 °C. The reaction was stopped with the addition of HBSS. Following this step, the bone marrow cells were centrifuged at 800× *g*, for 7 min at 4 °C; the supernatant was discharged; and the cells were cultivated in tissue culture-treated Petri dishes in RPMI 1640 medium supplemented with 15% *v*/*v* of L929 cell-conditioned medium (LCM), as a source of colony-stimulating factor-1 (CSF-1). The cells were cultivated at 37° C in an incubator with 5% CO_2_. After 24 h, the non-adherent cells were collected and cultured in sterile Petri dishes with R10 media supplemented with 15% *v*/*v* LCM for a week. On days 2, 3, 5, and 7, a further 15% of LCM was added to the culture. On day 8, the adherent cells were collected, counted, adjusted to 2 × 10^6^ macrophage/mL, and seeded in 96-well plates in round coverslips in 24-well plates in RPMI 1640 media supplemented with 10% fetal bovine serum, 10 μg/mL of gentamicin, and 100 U/mL of penicillin (R10).

### 2.5. Parasites

*Leishmania (L.) amazonensis* (MHOM/BR/1973/M2269) and *Leishmania (V.) braziliensis* (MHOM/BR/01/BA788) parasites were grown in Schneider’s Drosophila medium (Sigma-Aldrich Co., St. Louis, MO, USA), supplemented with 10% heat-inactivated fetal bovine serum (Gibco^®^; Thermo Fisher Scientific, Waltham, MA, USA), 10 μg/mL of gentamicin, and 100 U/mL of penicillin (S10), 25 °C. In all the experiments, promastigote forms in the stationary phase of growth were used.

### 2.6. Antipromastigote and Cytotoxic Assays

The promastigote forms of *L. (L.) amazonensis* and *L. (V.) braziliensis* were collected by centrifugation (1200× *g*, for 10 min at 4 °C), and the parasites were adjusted to 2 × 10^6^ parasite/well in a 96-well culture plate containing S10 medium at 25 °C. The parasites were incubated with serial dilutions of methanolic extract, alkaloid-enriched fraction, and dicentrine or miltefosine as a standard treatment (0.08 to 20 μg/mL). The control parasites were incubated with S10. After 24 and 72 h of incubation, the *L. (L.) amazonensis* and *L. (V.) braziliensis* parasites were centrifuged at 1200× *g*, for 10 min at 4 °C, and the supernatants were discarded and then washed three times with 200 μL of 1× PBS (pH 7.4). The parasite viability was then analyzed using resazurin (Thermo, USA). After 30 min of incubation, the viability was analyzed in an ELISA reader, using the following wavelength: 416 nm excitation and 574 nm emission. The concentration of the tested material that eliminates 50% of the parasites—effective concentration 50% (EC_50_)—was obtained by using correlation analysis in the GraphPad Prism 9.0 software.

The cytotoxic effects of the methanolic extract, alkaloid-enriched fraction, and dicentrine were analyzed in bone marrow-derived macrophages. These cells were adjusted to 10^6^ macrophages/well in a 96-well plate containing R10; twenty-four hours later, serial dilutions of methanolic extract, alkaloid-enriched fraction, dicentrine, or miltefosine (0.08 to 20 μg/mL) as standard treatment were added to the macrophages, which were maintained in a humidified incubator at 37 °C and 5% CO_2_. The macrophage toxicity was analyzed after 24 and 72 h of incubation with natural products. The cell viability was analyzed with resazurin, as described above. The concentrations that eliminate 50% of the cell population—cytotoxic concentration 50% (CC_50_)—were estimated using correlation analysis in GraphPad Prism 9.0 software. The selectivity indexes (SIs) of the tested plant materials and miltefosine were estimated by the ratio between the CC_50_ and EC_50_ [36].

### 2.7. Infection and Experimental Treatment (In Vitro)

Bone marrow-differentiated macrophages were cultured in round coverslips at the density of 10^5^ macrophage/coverslip for 2 h. After this period, the coverslips were added to 24-well plates, 500 μL of R10 media was added to each well, and the cells were cultured for 24 h. After this period, the macrophages were infected with the *L. (L.) amazonensis* or *L. (V.) braziliensis* promastigote forms in the stationary phase of growth at a ratio of 10 parasites per 1 macrophage [36,37] in a humidified incubator with 5% CO_2_ at 35 ° C for 24 h. To remove the non-internalized parasites, each well was washed three times with 1 mL of warm 1× PBS. Then, the methanolic extract or alkaloid-enriched fraction (2.5, 5.0, and 10 μg/mL), dicentrine (1.25, 2.5, and 5.0 μg/mL), or miltefosine (2.5, 5.0, 10.0, and 20.0 μg/mL) were added to the infected cells for 24 and 72 h. The infected controls were cultured with R10 medium only. At 24 and 72 h of incubation, the cells were washed three times with warm 1× PBS, and the coverslips were dried at room temperature, fixed with methanol, and stained by Giemsa (Sigma-Aldrich, USA). At least 200 cells/coverslip were quantified and natural product concentrations that decreased to 50% of the infection index (effective concentration 50%—EC_50_), were obtained by using correlation analysis in the GraphPad Prism 9.0 software.

### 2.8. Quantification of Hydrogen Peroxide and Nitric Oxide

The bone marrow-derived macrophages (10^5^ macrophage/well) were cultured in a 96-well black plate in R10 media in a humidified incubator containing 5% CO_2_ at 35 °C for 24 h. After this period, the macrophages were washed three times with warm 1× PBS and incubated with 1.25, 2.5, and 5 μg/mL of dicentrine for 24 or 72 h. The controls were incubated with R10 media only.

In another set of experiments, the macrophages were infected with the *L. (L.) amazonensis* or *L. (V.) braziliensis* promastigote forms (details in item 2.7). Twenty-four hours later, the cells were incubated with dicentrine as detailed above and the controls (infected and noninfected) with R10 only. In both sets of experiments, the positive controls were incubated with 100 ng/mL of lipopolysaccharide (Sigma-Aldrich, USA) for 24 and 72 h.

At each time point, the cells were washed three times with 200 μL of warm 1× PBS and then incubated with 5 μM of 4-amino-5-methylamino-2’,7’-difluorofluorescein diacetate (DAF-FM) for 60 min to analyze the intracellular nitric oxide (NO) production. The plates were read with 515 nm emission and 495 nm excitation. The H_2_O_2_ levels were quantified using the Amplex red kit (10-acetyl-3,7-dihydroxyphenoxazine), according to the manufacturer (Thermo Scientific, Waltham, MA, USA). Briefly, 50 μL of macrophage supernatants was mixed with 100 μM Amplex Red and 0.2 U/mL horseradish peroxidase. The reaction was incubated in the dark, at 25 °C for 30 min. A standard curve containing H_2_O_2_ was built in the range of 0.15 to 10 μM. The fluorescence intensity was measured in a plate reader with excitation at 544 nm and emission at 590 nm.

In both reactions, the blank controls, built with R10, R10 plus dicentrine, or LPS, did not interfere with the fluorescence spectra of the reaction.

### 2.9. Production of Dicentrine Formulation

Beeler’s base cream (10 g) was produced by melting the oily phase, which was composed of cetyl alcohol (1.5 g) and white wax (0.1 g) at 70 °C. The aqueous phase, manufactured with sodium lauryl sulfate (0.2 g), propylene glycol (1 g), butylhydroxytoluene (2 mg), and distilled water (q.s. 10 g), was then heated to 70 °C and mixed with the oily phase to produce the Beeler base cream. Purified dicentrine (50 mg) was dispersed in 50 µL of propylene glycol before being incorporated into Beeler’s base using a mortar and pestle. The blank and dicentrine-containing creams were packaged in amber glass bottles and no changes in the physical appearance of the creams were observed for six months at room temperature (~25 °C).

### 2.10. Characterization of Dicentrine Cream

The LUMiSizer dispersion analyzer (LUM, Berlin, Germany) was used to acquire the instability index of each cream. Aliquots of freshly prepared cream were transferred to a 2 mm path-length polycarbonate cuvette. To analyze physical stability, all the samples were added to a polycarbonate cuvette and all the samples were subjected to three centrifugation cycles at 1600× *g* at 25° C using the LUMiSizer. During the analysis, 300 optical profiles were acquired at intervals of 10, 30, and 50 s, employing a wavelength of 865 nm, totaling 66 min of analysis. The SEPView^®^ 6 software, integrated with the equipment, was used to perform a stability analysis based on the instability index.

### 2.11. Permeation Studies with Creams Containing Dicentrine

A Strat-M^®^ artificial membrane (Merck-Millipore, Burlington, MA, USA) was used to analyze the permeation profile of dicentrine, that is, an artificial membrane mimicking human skin [38]. Strat-M^®^ was mounted between the donor and receptor chamber of the Franz diffusion cells, containing a diffusion area of 1.766 cm^2^ with a total volume of 12 mL. The receptor chamber was filled with a mixture of phosphate buffer (pH 5.5) and dimethyl sulfoxide (1:1, *v*:*v*).

The donor compartment was filled with 1× PBS (pH 7.4) for 30 min, when the system reached 35 °C. After this period, the PBS was removed and 100 mg of cream containing 0.5% of pure dicentrine was added to the donor compartment in contact with the membrane. At the following time points, 5, 10, 15, 30, 45, 60, 120, 240, and 360 min, 1 mL samples were removed from the receptor chamber and stored at −20 °C for the HPLC analysis. At each time point, the receptor chamber was replenished with another 1 mL of prewarmed buffer. At the end of 360 min, the artificial membranes were wiped with a cotton bud impregnated with PBS to remove excess formulation. The membranes were weighed and homogenized with 2 mL of PBS with DMSO (1:1, *v*:*v*), vortexed, and centrifuged (10 min; 4000× *g*); the supernatants were collected and stored at −20 °C for the HPLC analysis.

The collected samples were analyzed using UHPLC (Ultimate 3000 standard Quaternary System). The integration of the peaks was performed using the Chromeleon 7.3.1.6535 program. An analytical Kinetex EVO 5 mm C18 reverse-phase HPLC column (150 × 4.6 mm) was used for the analysis. Isocratic elution was used with a mobile phase consisting of MeOH:H_2_O (7:3 *v*/*v*). The flow rate was set at 2 mL/min, while the injection volume was 10 μL. Detection was carried out at 220 nm, and a linear calibration curve was achieved between 0.1 and 100 μg/mL (R^2^ > 0.9987) of dicentrine (peak at retention time of 2.0 min). By using a standard curve built with dicentrine, a regression analysis was used to calculate the slopes and intercepts of the linear portion of each graph. The steady-state flux (J_SS_), the permeability coefficient (P), the diffusion coefficient, and the lag time were estimated as previously described by Lalatsa and collaborators [39].

### 2.12. Infection and Experimental Treatment

Thirty male BALB/c mice were infected at the base of the tail with 10^6^ promastigote forms of *L. (L.) amazonensis* in the stationary phase of growth. The control animals received PBS under the same route. Thirty days after infection, the infected animals were divided into four groups, as follows: Groups 1 and 2 constituted infected animals that were treated with 1.7 mg of cream containing 0.5% *w*/*w* of dicentrine per animal; Group 2 was treated with Beeler’s base cream (1.7 mg of cream/animal); Group 3 was treated with 5 mg/kg of deoxycholate amphotericin intraperitoneally per animal; and Group 4 was the infected control, injected intraperitoneally with 20 μL of PBS. Topical treatment in Groups 1 and 2 was performed at the base of the tail, with a surface area of approximately 5.2 mm^2^. All the animals were treated for 10 consecutive days, once daily. The size of the lesions was recorded once a week. Five days after the last dose, all the groups were euthanized with a lethal dose of thiopental. Skin fragments were collected to analyze tissue parasitism by a quantitative limiting dilution assay [40]. Briefly, a skin fragment from the base of the tail of each group was collected aseptically, weighed, and homogenized in S10 medium. The tissue suspensions were subjected to 12 serial dilutions with four replicate wells. The number of viable parasites was determined based on the highest dilution at which the promastigotes could grow after 10 days of incubation at 25° C.

### 2.13. Statistical Analysis

The experiments were repeated three times; the obtained results were presented as the mean ± standard error. The statistical analyses were performed using GraphPad Prism 9 software and the ANOVA test was used to analyze the differences between the groups. Statistical significance was established at *p* < 0.05.

## 3. Results

### 3.1. Leishmanicidal and Cytotoxic Properties of O. puberula

The methanolic extract, alkaloid-enriched fraction, and dicentrine, obtained from the leaves of *O. puberula*, were assayed in the promastigote forms of *L. (L.) amazonensis* and *L. (V.) braziliensis* as well as the main host cells of *Leishmania*, the macrophages.

The methanolic extract of the leaves of *O. puberula* was active on the *L. (L.) amazonensis* and *L. (V.) braziliensis* promastigote forms at 24 and 72 h of incubation (Table 1) and showed no toxic events to the macrophages, considering the highest concentration tested (20 μg/mL). Furthermore, it was verified that the methanolic extract was more selective to *L. (L.) amazonensis* than *L. (V.) braziliensis* at 72 h. The alkaloid-enriched fraction obtained from the methanolic extract showed potent leishmanicidal activities on *L. (L.) amazonensis* and *L. (V.) braziliensis* (Table 1). In *L. (L.) amazonensis*, it was 5.6 and 7.3 times more active at eliminating the promastigote forms than the methanolic extract. In *L. (V.) braziliensis*, it was 3.3 and 17.5 times more active in eliminating the promastigote forms than the methanolic extract, respectively (Table 1). On the macrophages, this fraction induced a CC_50_ of 14.2 ± 1.5 and 16.7 ± 0.3 μg/mL at 24 and 72 h, respectively (Table 1). The selective index was higher toward *L. (L.) amazonensis* at 24 h but lower at 72 h in comparison to *L. (V.) braziliensis.*

On the other hand, dicentrine was observed to show potent leishmanicidal activity in both *Leishmania* species at 24 and 72 h. On the macrophages, dicentrine was observed to induce a CC_50_ of 17.8 ± 1.1 μg/mL and greater than or equal to 20 μg/mL at 24 and 72 h, respectively (Table 1). Concerning the selective indexes, it was verified that dicentrine was more selective to *L. (L.) amazonensis* than *L. (V.) braziliensis*, especially after 72 h of incubation, based on the calculated selectivity indexes (Table 1).

On the contrary, miltefosine was found to be less active than the methanolic extract, fraction, and dicentrine in eliminating the promastigote forms of *L. (L.) amazonensis* and *L. (V.) braziliensis*; furthermore, the selective indexes of miltefosine at 24 h were 10.9 and 5.7 times less specific than the SI of dicentrine on the *L. (L.) amazonensis* and *L. (V.) braziliensis* promastigotes, respectively, and at least 30.3 and 11.1 times less active at 72 h compared to the dicentrine selective indexes, respectively (Table 1).

The methanolic extract of the leaves of *O. puberula* was active in the intracellular forms of *L. (L.) amazonensis* and *L. (V.) braziliensis* at 24 and 72 h of incubation (Table 2). Furthermore, it was verified that the methanolic extract was more active and selective toward *L. (L.) amazonensis* rather than the *L. (V.) braziliensis* amastigote forms.

The alkaloid-enriched fraction also exhibited potent leishmanicidal activities in the *L. (L.) amazonensis* and *L. (V.) braziliensis* amastigote forms (Table 2). However, leishmanicidal activities were not potentialized as the methanolic extract was fractionated, especially for *L. (L.) amazonensis*-infected macrophages incubated for 24 and 72 h with this fraction.

In *L. (L.) amazonensis*, it was observed that dicentrine was 2.0 and 2.8 times more active in eliminating the amastigote forms than the methanolic extract, while in *L. (V.) braziliensis*, it was 4.7 and 3.6 times more active in eliminating the amastigote forms than the methanolic extract, respectively (Table 2). The selective index induced by dicentrine was higher in *L. (L.) amazonensis* infection than in *L. (V.) braziliensis* (Table 2).

Miltefosine was less active and selective in *L. (L.) amazonensis* infection (*in vitro*) in comparison to the methanolic extract, the alkaloid-enriched fraction, and the dicentrine. In *L. (V.) braziliensis* infection, miltefosine exhibited activity similar to that of the methanolic extract only at 24 h; however, the alkaloid-enriched fraction and dicentrine were more active and selective than miltefosine (Table 2).

### 3.2. Quantification of Hydrogen Peroxide and Nitric Oxide Produced by Macrophages

**Figure 1:**
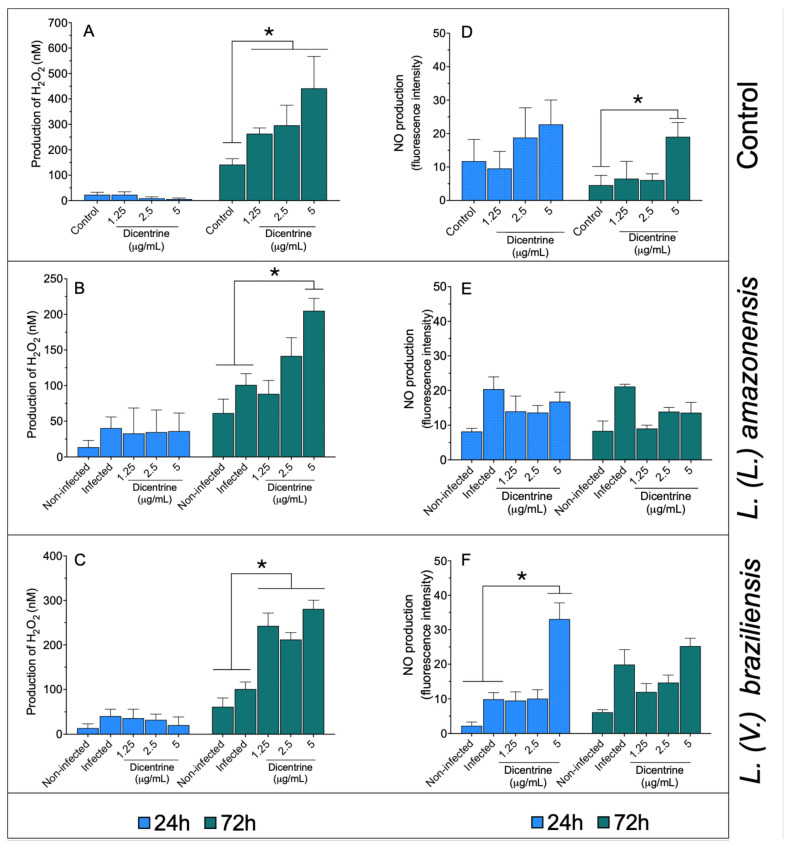
Infected and noninfected bone marrow-differentiated macrophages were treated with 1.25, 2.5, and 5 μg/mL of dicentrine purified from *O. puberula* leaves for 24 and 72 h, when H_2_O_2_ (**A**–**C**) and NO (**D**–**F**) levels were quantified. * *p* < 0.05 in comparison to controls.

Noninfected macrophages (Figure 1A) treated with 1.25, 2.5, and 5.0 μg/mL of dicentrine showed increased levels of hydrogen peroxide compared to the control after 72 h of incubation (*p* < 0.05). In a similar manner, it was observed that *L. (L.) amazonensis-* (Figure 1B) and *L. (V.) braziliensis*-infected macrophages (Figure 1C) treated with 1.25, 2.5, or 5.0 μg/mL of dicentrine produced significant levels of this reactive oxygen metabolite compared to the noninfected and infected groups (*p* < 0.05).

In the macrophages, dicentrine did not cause an increase in NO levels at 24 h; however, 5 μg/mL of this compound was able to increase the NO levels compared to the control at 72 h of incubation (Figure 1D). The macrophages infected with *L. (L.) amazonensis* (Figure 1E) and treated with dicentrine did not change the NO production after 24 or 72 h of incubation compared to the controls. In the macrophages infected with *L. (V.) braziliensis* (Figure 1F) and treated with 5 μg/mL of dicentrine for 24 h, a significant increase in the NO level was observed compared to the control, but at 72 h, only basal levels of NO were recorded in *L. (V.) braziliensis* infection.

### 3.3. Stability, Sedimentation Velocity, and Permeation of a Topical Formulation Containing Dicentrine

Taking into account the potent leishmanicidal property of purified dicentrine, a cream containing 0.5% of this alkaloid was formulated. The physical stability of the cream was analyzed to verify a tendency of phase separation, which is one of the main indicators of emulsion stability. In this regard, Beeler’s basis (blank cream) was observed to show an instability index of 0.024 ± 0.002, suggesting reasonable stability under the tested conditions (Table 3). After the addition of alkaloid, no significant changes were observed in the dicentrine cream (Table 3), which presented an instability index of 0.022 ± 0.003. This result suggests that all the formulations exhibited high stability. Regarding the sedimentation velocity (Table 3), it was observed that the Beeler basis showed a sedimentation velocity of 4.357 ± 0.035 mm/s, while the addition of dicentrine in the Beeler basis led to a significant increase (*p* < 0.05) of 19.8% of the sedimentation velocity (5.221 ± 0.056 mm/s).

Cutaneous permeation studies using the Strat-M membrane showed that dicentrine did not permeate through the artificial membrane within the six hours of experiments; however, it was retained in the membrane with a concentration of 1.96 ± 0.37 μg of dicentrine per mg of membrane.

### 3.4. Experimental Treatment

The BALB/c mice infected with *L. (L.) amazonensis* were treated with 0.5% *w*/*w* of dicentrine or blank creams by the topical route or 5 mg/kg of deoxycholate amphotericin B by the intraperitoneal route once a day for 10 days. In this case, it was observed that the blank cream did not change the progression of the lesion. However, the cream containing 0.5% *w*/*w* of dicentrine reduced the progression of the lesion in the last week of treatment by 23.8% (*p* < 0.05). The animals treated with amphotericin B intraperitoneally exhibited a significant reduction in the lesion at the fifth and sixth weeks after infection compared to the control (*p* < 0.05).

**Figure 2:**
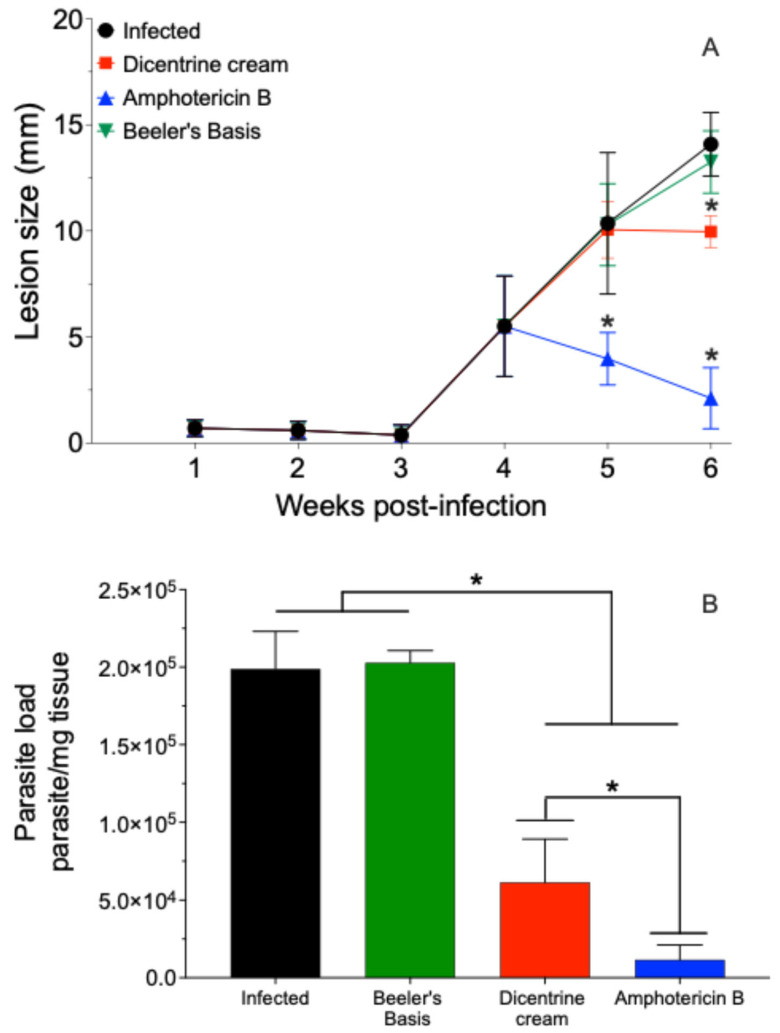
BALB/c mice were infected at the base of the tail with 10^6^ *L. (L.) amazonensis* promastigote forms in stationary phase of growth. After 4 weeks, topical treatment with cream containing 0.5% *w*/*w* dicentrine (1.7 mg/dose), blank cream (1.7 mg/dose), or intraperitoneally with amphotericin (5 mg/kg) was started. The animals were treated once a day for 10 days. The development of the lesion was monitored during 6th weeks PI with a micrometer (**A**). The parasite load in the skin was analyzed by limiting-dilution assay (**B**). * *p* < 0.05 in comparison to the infected group.

The infected animals and animals treated with the blank cream exhibited tissue parasite burdens of 198,863.3 ± 24,194.3 and 202,815.7 ± 7821.9 parasite/mg of skin, respectively (Figure 2B). In contrast, the animals treated with 0.5% *w*/*w* dicentrine cream resulted in a significant reduction in parasitism of 69.1% (61,489.1 ± 13,834.1 parasite/mg of skin) compared to the infected control. The animals treated with intraperitoneal amphotericin B showed a significant reduction in skin parasitism (2248.6 ± 1010.5 parasite/mg of skin) compared to the infected, blank, and dicentrine cream groups.

## 4. Discussion

The members of the Lauraceae family have been studied in different aspects of pharmacology; however, few studies have been performed with *O. puberula* despite the bioactive alkaloids that this plant biosynthesizes. Thus, this pionner study showed the leishmanicidal and immunomodulatory properties of the methanolic extract, an alkaloid-enriched fraction, as well as purified dicentrine, obtained from the O. puberula leaves on parasite species able to cause cutaneous leishmaniasis in Latin American (in vitro and in vivo).

*In vitro* studies showed that the methanolic extract was active in the promastigote and amastigote forms of *L. (L.) amazonensis* and *L. (V.) braziliensis* at 24 and 72 h of treatment without causing significant toxic events to the macrophages. Furthermore, the methanolic extract was more active in the promastigote and amastigote forms than miltefosine, the drug used in the therapy. Previous studies carried out with the methanolic extract produced with the leaves of *O. macrophylla* or with the leaves and barks of *O. duckei* showed leishmanicidal activity in *L. (L.) amazonensis*, *L. (L.) chagasi, L. (V.) braziliensis*, and *L. (V.) panamensis* promastigotes. However, the bioactivities of these extracts in promastigote forms were less active [41,42] than the one produced with *O. puberula*, probably due to the different chemical composition of each tested extract. Similarly, the cytotoxicity of the methanolic extract produced with *O. macrophylla* was low, giving a selectivity index of 2 [41], but in the present study, the SI was higher than the SI of *O. macrophylla,* in the range of 2.7 to 6.6 in *L. (L.) amazonensis* and 3.4–5.7 in *L. (V.) braziliensis*, suggesting that the compounds produced and accumulated by *O. puberula* are more selective than in other species.

Compared to the methanolic extract, the alkaloid-enriched fraction was more active on the promastigote of *L. (L.) amazonensis* and *L. (V.) braziliensis* parasites; but in the amastigote forms, the fraction tends to present an activity similar to that of the extract. The promastigote forms of both species were similarly affected by this fraction—in contrast, the *L. (V.) braziliensis* intracellular amastigote tended to be more resistant to this fraction than *L. (L.) amazonensis*. In macrophages, the alkaloid-enriched fraction was moderately toxic; however, the selectivity was moderate in the promastigote forms of *L. (L.) amazonensis* and *L. (V.) braziliensis,* always about 7.9, higher than the selectivity of miltefosine, suggesting that the increase in cytotoxicity does not interfere with the efficacy in the parasite. Possibly, the potent leishmanicidal activity of the alkaloid-enriched fraction should be related to the presence of dicentrine, the main alkaloid in this fraction, as well as the minoritarian alkaloids dicentrine-β-N-oxide, dehydrodicentrine, predicentrine, N-methyllaurotetanine, and cassythicine, as previously demonstrated [27]; moreover, the yield of these alkaloids was too low (0.01–0.04% relative to the methanolic extract) to purify them and perform further experiments.

In fact, after purification of the main alkaloid, dicentrine, from the alkaloid-enriched fraction, a potent leishmanicidal activity was observed mainly in the *L. (L.) amazonensis* promastigote forms at 24 and 72 h. Furthermore, dicentrine showed moderate cytotoxicity in the bone marrow-derived macrophages, which was already detected in another study [32]. Despite this activity, the selective index of dicentrine was higher in the promastigote forms of *L. (L.) amzonensis* (29.6–66.7) and *L. (V.) braziliensis* (14.8–33.3) compared to miltefosine (2.2–3.0). Similar behavior was observed for the intracellular amastigote forms of *L. (L.) amazonensis* and *L. (V.) braziliensis,* in which dicentrine was more selective in killing amastigotes than the alkaloid-enriched fraction and miltefosine. In this regard, at 24 h and 72 h, dicentrine was 7.1 and 15.7 times more active than miltefosine at eliminating *L. (L.) amazonensis*, respectively, and 5.9 and 10.9 times more effective than miltefosine in killing *L. (V.) braziliensis*. Furthermore, the selective index of dicentrine was higher than the SI of miltefosine, suggesting that this alkaloid should indeed be considered as an alternative drug for leishmaniasis. A previously published article showed that dicentrine from *O. puberula* was capable of killing the amastigote forms of *L. (L.) infantum* with an EC50 of ~3.5 μg/mL, while miltefosine had an EC50 of 4.3 μg/mL [32], reinforcing that dicentrine is more active in killing species of *Leishmania* that cause cutaneous disease. Nevertheless, these reports suggest that this alkaloid is active on parasite species that cause cutaneous and visceral leishmaniasis.

Considering the high activity and selectivity of dicentrine in the intracellular amastigotes of *L. (L.) amazonensis* and *L. (V.) braziliensis*, experiments related to macrophage stimulation were performed. In this case, it was observed that dicentrine mainly triggered the production of hydrogen peroxide and nitric oxide at 72 h of incubation in noninfected macrophages. During the infection with *L. (L.) amazonensis* or *L. (V.) braziliensis*, the levels of hydrogen peroxide remained high in comparison to the noninfected and infected controls, suggesting that this compound can kill parasites by triggering hydrogen peroxide production.

However, it is still important to note that, for example, 5 μg/mL of dicentrine induced 441 nM of hydrogen peroxide in the macrophage; however, *L. (L.) amazonensis-* or *L. (V.) braziliensis*-infected macrophages treated with the same concentration of dicentrine reduced the levels of this metabolite by almost two times. This fact suggests that both parasites have potent antioxidant enzymatic machinery associated with the presence of tryparedoxin peroxidase and catalase, which can inhibit the production of nitric oxide and/or hydrogen peroxide [43,44,45]. The presence of these antioxidant enzymes should be responsible for completely abrogating the production of nitric oxide in *L. (L.) amazonensis* infection, considering that tryparedoxin peroxidase has been elected as a scavenger enzyme and a virulence factor in *L. (L.) amazonensis* infection [44]. In contrast, in *L. (V.) braziliensis* infection, a significant increase in the nitric oxide levels was observed in the macrophages treated with 5 μg/mL of dicentrine at 24 h, suggesting an early and potent immunomodulatory activity of this compound, but after 72 h of treatment, the nitric oxide levels were similar to the controls. On the other hand, the immunomodulatory activity on macrophages may be shared by some alkaloids, such as mahanine isolated from *Murraya koenigii* leaves (Rutaceae), skimmianine from the fruit of *Spiranthera odoratissima* (Rutaceae), and berberine chloride, which was able to stimulate *Leishmania*-infected macrophages to produce nitric oxide and/or hydrogen peroxide metabolites, accounting for a significant reduction in intracellular parasitism [46,47,48]. Therefore, the leishmanicidal effect of dicentrine on *L. (L.) amazonensis* and *L. (V.) braziliensis* can be related to the immunomodulatory effect of this compound in phagocytic cells.

Considering that dicentrine was selective and exhibited immunomodulatory activity on infected macrophages, a topical formulation was produced and tested in the experimental model of cutaneous leishmaniasis. The emulsion was stable, and the Beeler base was observed to show a low instability index. The cream containing 0.5% *w*/*w* dicentrine showed a similar index compared to the Beeler base, suggesting that the addition of this bioactive compound does not alter emulsion stability with only moderate changes in the sedimentation rate [49,50]. This suggests that dicentrine cream could be an alternative to treat cutaneous leishmaniasis. Furthermore, studies related to dicentrine permeation, conducted for 6 hours, showed that this alkaloid was not able to permeate through the artificial membrane; however, dicentrine was impregnated into the membrane, with a concentration of 1.96 μg at the end of a 6 h experiment. The amount of dicentrine retained in the membrane represents at least double the dicentrine EC_50_ values, able to eliminate the *L. (L.) amazonensis* and *L. (V.) braziliensis* amastigote forms (*in vitro*), as shown in Table 2.

Therefore, the efficacy of 0.5% dicentrine cream was evaluated in the murine model of leishmaniasis caused by *L. (L.) amazonensis*. In terms of the lesion, at the end of the treatment, the dicentrine cream was observed to reduce the size of the skin lesions by 23.8% compared to the control and the animals treated with Beeler cream. On the other hand, systemic treatment with amphotericin B deoxycholate reduced lesion development by 80%. In addition to the reduction in the lesion, the dicentrine cream was found to cause a reduction in the number of amastigote forms in the skin (~69.1%), which corroborates that the amount of dicentrine retained in the artificial membrane (~1.96 μg), in fact, should be enough to eliminate more than 50% of parasites.

Previous studies have already shown that some plants that produce and accumulate some types of alkaloids have leishmanicidal activity. In this case, it was demonstrated that an alkaloid-enriched fraction from the trunk bark of *Aspidosperma nitidum* (Apocynaceae) given by oral route to BALB/c mice infected with *L. (L.) amazonensis* decreased the size of the cutaneous lesion, as well as the tissue parasitism [51]. Alkaloids produced by *Raputia heptaphylla* (Rutaceae), such as raputiolide and N-methyl-8-methoxyflindersine, also presented leishmanicidal activity *in vitro* [52], and the synthetic analogs carboxylic acid 2-ethyl-3-propyl-4-quinolinine and 2-amino-8-hydroxyquinoline given topically as an ointment at 1% improved the signs of cutaneous leishmaniasis caused by *L. (V.) panamensis* in hamsters, without side effects [53]. The alkaloid mahanine from Murraya koenigii (Rutaceae) given orally to BALB/c mice infected with L. (L.) donovani was able to visceral parasitism in these animals [46]. Therefore, it is possible to assume that some classes of alkaloids may be interesting targets for the development of new alternative drugs to treat leishmaniasis.

In the present study, it is still important to note that systemic amphotericin B deoxycholate treatment was more effective in reducing the size of the lesions and skin parasite load compared to topical treatment with dicentrine cream; however, amphotericin B as a systemic treatment causes severe side effects in patients, leading to the abandonment of therapy [12,54]. Furthermore, it is important to note that after the last injection of amphotericin B, the animals received a total dose of 1.25 mg of amphotericin B, which was able to decrease the parasitism by 98.8%. On the contrary, the animals treated locally with dicentrine cream received only a total accumulated dose of 0.085 mg of dicentrine, which was 14.7 times lower compared to the amphotericin B and still caused a reduction of 69.1% in skin parasitism.

## 5. Conclusions

In conclusion, it was observed that the methanolic extract and an alkaloid-enriched fraction of the leaves of *O. puberula* eliminated the promastigote and amastigote forms of *L. (L.) amazonensis* and *L. (V.) braziliensis*, without causing significant cytotoxicity to the macrophages. Furthermore, the main alkaloid, dicentrine, was highly active and selective in eliminating intracellular *L. (L.) amazonensis* and *L. (V.) braziliensis* parasites, mainly associated with the induction of H_2_O_2_. A cream containing 0.5% dicentrine reduced the progression of the size of the lesion and the skin parasitism. Finally, it is important to note that this is the first study to show that dicentrine formulated as a topical treatment has *in vivo* activity. This is the first step toward the development of therapeutic alternatives using dicentrine for the topical treatment of cutaneous leishmaniasis, especially for patients living in remote areas.

## Figures and Tables

**Table 1:** Antipromastigote and cytotoxic effects of the methanolic extract, alkaloid-enriched fraction, and purified dicentrine. Selective indexes were presented between parenthesis in bold. Data are presented as mean and standard deviation.

Plant Component	EC_50_^a^ (μg/mL)				CC_50_^b^ (μg/mL)	
	*L. (L.) amazonensis*		*L. (V.) braziliensis*		Macrophages	
	24 h	72 h	24 h	72 h	24 h	72 h
Methanolic extract	7.3 ± 2.5 **(≥2.7)**	2.9 ± 1.6 **(≥6.9)**	5.9 ± 0.8 **(≥3.4)**	3.5 ± 0.7 **(≥5.7)**	≥20	≥20
Alkaloid-enriched fraction	1.3 ± 0.3 **(10.9)**	0.4 ± 0.07 **(41.8)**	1.8 ± 0.2 **(7.9)**	0.2 ± 0.03 **(83.5)**	14.2 ± 1.5	16.7 ± 0.3
Dicentrine	0.6 ± 0.2 **(29.6)**	0.3 ± 0.06 **(≥66.7)**	1.2 ± 0.1 **(14.8)**	0.6 ± 0.1 **(≥33.3)**	17.8 ± 1.1	>20
Miltefosine	9.9 ± 0.6 **(2.7)**	18.5 ± 0.1 **(2.2)**	10.1 ± 1.2 **(2.6)**	13.9 ± 1.2 **(3.0)**	26.6 ± 1.4	41.5 ± 0.6

^a^ Effective concentration 50% (EC_50_); ^b^ cytotoxic concentration 50% (CC_50_).

**Table 2:** Anti-amastigote activity of the methanolic extract, alkaloid-enriched fraction, and purified dicentrine. Selective indexes were presented between parentheses in bold. Data are presented as mean and standard deviation.

	EC_50_ (μg/mL)			
Plant Component	*L. (L.) amazonensis*		*L. (V.) braziliensis*	
	24 h	72 h	24 h	72 h
Methanolic extract	1.9 ± 0.3 **(≥10.5)**	1.7 ± 0.5 **(≥11.8)**	4.6 ± 0.8 **(≥4.3)**	2.9 ± 0.7 **(≥6.9)**
Alkaloid-enriched fraction	1.3 ± 0.1 **(10.9)**	1.4 ± 0.4 **(11.9)**	3.5 ± 0.2 **(4.1)**	1.5 ± 0.03 **(11.1)**
Dicentrine	0.96 ± 0.5 **(18.5)**	0.6 ± 0.01 **(≥33.3)**	0.97 ± 0.1 **(18.3)**	0.8 ± 0.4 **(≥25.0)**
Miltefosine	6.8 ± 0.1 **(3.9)**	9.4 ± 0.9 **(4.4)**	5.7 ± 1.8 **(4.7)**	8.7 ± 1.2 **(4.8)**

**Table 3:** Instability indexes and sedimentation velocities were analyzed in Beeler’s basis and dicentrine cream. * *p* < 0.05 in comparison to Beeler’s basis.

Sample	Instability Index	Sedimentation Rate (mm/s)
Beeler’s basis	0.024 ± 0.002	4.357 ± 0.035
Dicentrine cream	0.022 ± 0.003	5.221 ± 0.056 *

## Data Availability

The original contributions presented in this study are included in the article. Further inquiries can be directed to the corresponding author.
